# A Serological Multiplexed Immunoassay (MIA) Detects Antibody Reactivity to SARS-CoV-2 and Other Viral Pathogens in Liberia and Is Configurable as a Multiplexed Inhibition Test (MINT)

**DOI:** 10.3390/immuno4010007

**Published:** 2024-03-03

**Authors:** Brien K. Haun, Albert To, Caitlin A. Williams, Aquena Ball, Karalyn Fong, Teri Ann S. Wong, Bode Shobayo, Julius Teahton, Lauren Ching, Varney Kamara, Davidetta M. Tekah, Peter Humphrey, John Berestecky, Vivek R. Nerurkar, Axel T. Lehrer

**Affiliations:** 1 Department of Cell and Molecular Biology, John A. Burns School of Medicine, University of Hawaii at Manoa, Honolulu, HI 96813, USA; 2 Department of Tropical Medicine, Medical Microbiology and Pharmacology, John A Burns School of Medicine, University of Hawaii at Manoa, Honolulu, HI 96813, USA; 3 National Public Health Institute of Liberia, Monrovia 1000, Liberia; 4 Department of Biological Sciences, Medical Science, TJR Faulkner College of Science and Technology, University of Liberia, Fendall 1000, Liberia; 5 Math Science Department, Kapiolani Community College, University of Hawaii, Honolulu, HI 96816, USA

**Keywords:** SARS-CoV-2, hCoV-NL63, DENV-2, CHIKV, serological, surveillance, inhibition, antibodies, Liberia, West-Africa

## Abstract

The SARS-CoV-2 pandemic ignited global efforts to rapidly develop testing, therapeutics, and vaccines. However, the rewards of these efforts were slow to reach many low- to middle-income countries (LMIC) across the African continent and globally. Therefore, two bead-based multiplexed serological assays were developed to determine SARS-CoV-2 exposure across four counties in Liberia. This study was conducted during the summer of 2021 on 189 samples collected throughout Grand Bassa, Bong, Margibi, and Montserrado counties. Our multiplexed immunoassay (MIA) detected elevated exposure to SARS-CoV-2 and multiple variant antigens. Additionally, we detected evidence of exposure to Dengue virus serotype 2, Chikungunya virus, and the seasonal coronavirus NL63. Our multiplexed inhibition test (MINT) was developed from the MIA to observe antibody-mediated inhibition of SARS-CoV-2 spike protein binding to its cognate cellular receptor ACE-2. We detected inhibitory antibodies in the tested Liberian samples, which were collectively consistent with a convalescent serological profile. These complementary assays serve to supplement existing serological testing needs and may enhance the technical capacity of scientifically underrepresented regions globally.

## 1. Introduction

The Severe Acute Respiratory Syndrome Coronavirus-2 (SARS-CoV-2) has caused a global health crisis since its discovery in December 2019. The disease it causes, known as Coronavirus Disease-19 (COVID-19), created a surge in research and development for tests, vaccines, and treatments. Reverse-transcription polymerase chain reaction (RT-PCR) and antigen-based tests quickly became available to the public and were utilized to curtail the spread of COVID-19. However, the needs of much of Africa were neglected as high-income countries received the majority of critical testing kits and supplies, while African LMICs received little despite having the expertise and facilities for running them [1]. Liberia, located in West Africa, reported 7504 cases and 294 deaths as of 2022 July 15 [2]. While case numbers have increased to 8090, there have been no new reports of COVID-19-related deaths (accessed 2023 August 8) [2]. Considering the population of Liberia was last reported to be 5.058 million people, these numbers likely underrepresent the country’s COVID-19 burden.

For clinical applications, RT-PCR tests are a reliable standard for COVID-19 testing to detect active infection. However, PCR testing requires substantial amounts of specialized resources to test large populations effectively and requires separate reactions to identify variant viral strains. Additionally, the reagents required for RT-PCR tests are sensitive to freeze–thaw conditions frequently incurred in LMICs. The purification and storage of RNA may also be similarly affected. It is possible that these limitations in resources have resulted in reduced COVID-19 testing and reporting in LMICs.

More approachable antigen-based tests, such as lateral flow tests, have been developed for at-home use [3,4]. These antibody-based antigen capture tests are rapid and can be easily scalable [5,6]. Lateral flow tests could be distributed globally, without cold-chain, but largely lack reliability and cannot distinguish between viral variants [6]. Serological tests, such as enzyme-linked immunosorbent assays (ELISA), allow anti-viral serological IgG detection and require less temperature-dependent reagents, enabling comprehensive testing in remote areas. However, ELISA requires separate reactions to detect reactivity to different antigens, requiring more antigen and serum for serological surveys spanning a larger panel of viral targets.

In non-clinical serological testing scenarios, bead-based systems, such as the Magplex^®^ platform (Luminex Corporation, Austin, TX, USA), offer flexibility in the number of antigen-antibody reactions that can be detected in a single well of a 96-well plate while maintaining high sensitivity and specificity [7]. Assays described as multiplexed immunoassays (MIAs) are ideal for serological surveys and can quickly identify exposure to several antigens [8–13]. The ability to test small amounts of serum or plasma samples against many antigenic targets at once limits the amount of time and materials needed to accomplish large-scale serological surveys. Furthermore, the equipment required to run an MIA can be easily transported to regions with limited infrastructure [9]. These unique characteristics make MIAs ideal for field studies and implementation where viral serological surveillance is needed, and resources may be scarce. However, these assays serve to assess prior exposure rather than active infection.

A critical component of serological surveillance is the assessment of the potential for antibody-mediated protective efficacy, specifically, neutralization against certain viruses. Wild-type SARS-CoV-2 assays require a biosafety level-3 (BSL-3) containment facility, which is not available in many regions of the world. Traditionally, plaque reduction neutralization tests (PRNT) are performed to report an individual’s 50%, 80%, or 90% neutralization titer. The COVID-19 pandemic sparked the development of surrogate neutralization tests that can be conducted under less stringent biosafety conditions to ameliorate the scarcity of the reported wild-type SARS-CoV-2 PRNT titers [14–17]. These inhibition assays are based on modified ELISA technology and measure the ability of serum antibodies to inhibit the binding of the SARS-CoV-2 spike protein to its cellular receptor, the angiotensin-converting enzyme-2 (ACE-2), which enables viral entry into host cells. Unfortunately, ELISA-based systems only allow inhibition to be tested against one SARS-CoV-2 variant at a time. A multiplexed surrogate neutralization assay should be able to detect the inhibition of receptor-binding of multiple virus variants to better characterize the previous exposure of individuals to SARS-CoV-2 or related viruses utilizing the same receptor-binding mechanism for entry while preserving sample volume.

In this study, we developed and implemented an MIA to document the serum reactivity to a multitude of antigens in samples collected from four counties in Liberia. While SARS-CoV-2 exposure was the primary focus, we included antigens from viruses previously underreported despite their suspected abundant presence in West Africa. Our panel consisted of antigens to SARS-CoV-2, hCoV-NL63 (NL63), Dengue virus serotype 2 (DENV-2), and Chikungunya virus (CHIKV). We confirmed assay performance using naïve and convalescent sera from samples collected in Hawaii and showed that the assay could further distinguish IgG levels between vaccine doses one and two. We determined that the samples from Montserrado, Margibi, Bong, and Grand Bassa counties show high rates of SARS-CoV-2 seroreactivity despite the low number of cases reported based on RT-PCR positivity. We additionally see evidence of exposure to the seasonal coronavirus NL63 and the mosquito-borne DENV-2 and CHIKV. Lastly, we developed and implemented a multiplexed inhibition test (MINT) for SARS-CoV-2 and its variants, which is adaptable to the MIA panel.

## 2. Materials and Methods

### 2.1. Sample Collection and Transportation

Samples were previously collected, with informed consent, from study participants in Hawaii [18] and four counties of Liberia (June of 2021) under IRB protocols approved by the University of Hawaii IRB and by the National Research Ethics Board of Liberia (NREB). All work was performed in accordance with institutional and governmental guidelines and regulations. Two of the Liberian counties, Bong and Grand Bassa, are known epicenters for Lassa fever in Liberia. In contrast, the other two, Montserrado and Margibi, were hotspots for Ebola Virus Disease (EVD) during the outbreak of 2014/2015. Approximately 4–5 milliliters (mL) of whole blood were aseptically collected through venipuncture into serum collection tubes (Frank Healthcare Co. Ltd., Wenzhou, China) from 206 study participants. After collection and clotting, blood samples were centrifuged at 1600× *g* for 10 min to separate the serum, which was aliquoted into appropriately labeled 1.5 mL screw-cap tubes. Serum tubes were immediately frozen at −80 °C and transported to the University of Liberia Fendall Laboratory within 24 h to be stored until further analysis.

### 2.2. Protein Expression and Purification

All plasmids, unless otherwise stated, were generated by inserting *Drosophila* codon-optimized, synthetic gBlock^™^ Gene Fragments (IDT, Coralville, IA, USA) downstream of an inducible metallothionine promoter and BiP secretion signal (pMT-BiP, Invitrogen, Waltham, WA, USA). The Coomassie stained sodium dodecyl sulfate-polyacrylamide gel electrophoresis (SDS-PAGE) and Western blots of each purified protein are shown in Supplemental Figure S1A–D.

#### 2.2.1. Human ACE-2 (hACE-2)

The gene encoding a soluble, truncated human angiotensin-converting enzyme-2 (aa 21–614, accession number: NP_001373188.1) was designed with restriction enzyme cleavage sites flanking the 3′ and 5′ ends, and with a C-terminal 6x histidine-tag. The gBlock^™^ was digested with restriction enzymes and ligated using T4 ligase (NEB, Ipswich, MA, USA) into pMT-BiP. The resulting plasmid and a pCoHygro selection plasmid were then co-transfected into *Drosophila* S2 cells using the Lipofectamine LTX with PLUS reagent (Invitrogen, Carlsbad, CA, USA) according to the manufacturer’s instructions. Stably transformed cell lines were created by selection with culture medium containing hygromycin B at 300 μg/mL for four weeks. Expression of hACE-2 from the cell line was induced by addition of 200 μM CuSO_4_ into the cell culture for one week at 26 °C. Recombinant hACE-2 was purified from clarified cell culture supernatants by nickel affinity chromatography (IMAC) using an Akta Pure FPLC with a Histrap FF column (Cytiva, Marlborough, VA, USA) equilibrated with PBS containing 300 mM NaCl + 0.01% Na azide. The loaded column was first washed to baseline using equilibration buffer containing 10 mM imidazole and eluted into a fraction collector with a linear gradient up to 50 mM, followed by a step elution of 200 mM imidazole in equilibration buffer. The purified fractions were buffer exchanged into PBS using a 30 kDa MWCO Amicon Ultra centrifugal filter unit (Millipore Sigma, Burlington, MA, USA). The purity and immunoreactivity of the protein was evaluated by SDS-PAGE and western blotting using a 6x-his-tag secondary antibody (Thermo Fisher Scientific, Waltham, WA, USA).

#### 2.2.2. SARS-CoV-2 S Trimer

A codon-optimized sequence encoding a furin cleavage-resistant, transmembrane-deleted spike (S) glycoprotein of SARS-CoV-2, Wuhan-Hu-1 strain, (aa 12–1147, accession number NC_045512) with a C-terminal T4-foldon trimerization domain, was used to express the Wu-1 trimer. The plasmid construct of Wu-1 Trimer was assembled from four clonal gBlock^™^ fragments (IDT, Coralville, IA, USA) using a HIFI Assembly kit (NEB, Ipswich, MA, USA) into pMT-BiP according to the manufacturer’s instructions. Two additional proline substitutions (K986P and V987P) between the heptad repeat 1 and central helix regions, as well as the removal of the S2′ protease cleavage site (K814G and R815A) were also incorporated into the design of the gene. Correctly assembled plasmids were screened using Sanger sequencing and selected plasmids were used for transfecting Drosophila S2 cells to generate stably expressing cell lines as described above. Recombinant S proteins were purified from clarified cell culture supernatants by immunoaffinity chromatography (IAC) using NHS-activated Sepharose (Cytiva, Marlborough, MA, USA) coupled with mAb CR3022. Bound recombinant S protein was washed with PBS + 0.02% Tween20 and PBS before being eluted with an isocratic step of 20 mM glycine buffer, pH 2.5. The purified Wu-1 Trimer was concentrated, buffer-exchanged into PBS, and analyzed by SDS-PAGE and Western blotting using mAb CR3022 as the detection antibody. Antigens were quantified and stored at −80 °C.

#### 2.2.3. SARS-CoV-2 NP

The full-length SARS-CoV-2 NP (Wu-1 NP) coding region (aa 1–419, Accession number NC_045512.2) was cloned from a commercial plasmid containing the NP gene (IDT, Coralville, IA, USA) using Q5 High-Fidelity polymerase (NEB, Ipswich, MA) with gene-specific primers (5′-ATGCGGATCCATGTCTGATAATGGACCCCAAAATCAGCGAAATGCACCCCGCATTACG-3′ and 5′-ACGTACGTCGACGGCCTGAGTTGAGTCAGCACTGCTCATGGATTGTTGCAATTGTT TG-3′). A PCR protocol recommended by the manufacturer was followed and included a denaturization temperature (temp) of 98 °C for 10 s, an annealing temp of 55 °C for 30 s, and an elongation temp of 72 °C for 90 s, for 30 cycles. The resulting PCR product was digested with restriction enzymes and ligated into pMT-BiP upstream of the 6x his-tag sequence as described above. The transfection, generation of stably transformed cell lines, and verification of protein expression were also carried out as described above. Recombinant Wu-1 NP was purified from the cell culture supernatant using a HisTrap excel column (Cytiva, Marlborough, VA, USA). The protein was eluted with two-step gradients of 50 mM and 75 mM imidazole in equilibration buffer, followed by two linear gradients up to 150 mM and 500 mM imidazole. Fractions containing the purified Wu-1 NP were buffer-exchanged into PBS and stored at −80 °C. The immunoreactivity of the protein was evaluated by SDS-PAGE and Western blot using human SARS-CoV-2 convalescent sera (1:500 dilution) as the detection antibody.

#### 2.2.4. CHIKV E2

A codon-optimized, synthetic gene encoding a truncated CHIKV E2 gene from the Chikungunya strain Senegal 37997 (aa 339–689, Accession number Q5XXP3.1), flanked with restriction enzyme sites, was used as the gene cassette. The generation of plasmids, transfection, production of stably transformed cell lines, and verification of protein expression were carried out as described above. Recombinant CHIKV E2 protein was purified from clarified cell culture supernatants with a HisTrap FF column (Cytiva, Marlborough, MA, USA) using two step elutions of 100 mM and 150 mM imidazole and two linear gradients to 350 mM and 500 mM of imidazole in equilibration buffer. Purified CHIKV E2 was concentrated, and buffer exchanged into PBS, quantified, and stored at −80 °C. The immunoreactivity of the protein was evaluated by SDS-PAGE and Western blot using human CHIKV-convalescent sera (1:500 dilution) as the detection antibody.

### 2.3. Coupling of Microspheres with Recombinant Antigens

The coupling of individually addressable microspheres with Wu-1 Trimer, Wu-1 S1 (SinoBiological 40591-V08H), Alpha S1 (SinoBiological 40591-V08H7), Beta S1 + S2 (SinoBiological 40589-v08B16), Wu-1 NP, NL63 S1 (SinoBiological 40600-V08H), DENV-2 E (SinoBiological 40471-V08Y1), DENV-2 NS1 (Native Antigen Company, Oxford, UK), and CHIKV E2 proteins, was conducted as described previously [9,10,13]. Briefly, 25μg of each antigen were coupled to 2.5 × 10^6^ microspheres in a two-step carbodiimide process following the manufacturer’s recommendation (Luminex Corporation, Austin, TX, USA). Microspheres dyed with varying amounts of a spectrally different fluorophore were also coupled with bovine serum albumin as controls. Internally dyed, carboxylated, magnetic microspheres (Mag-Plex^™^-C) were obtained from Luminex Corporation (Austin, TX, USA).

### 2.4. Microsphere Immunoassay (MIA)

Human IgG was detected as previously described [9,10,13]. Briefly, microspheres coupled with Wu-1 Trimer, Wu-1 S1, Alpha S1, Beta S1 + S2, Wu-1 NP, NL63 S1, DENV-2 E, DENV-2 NS1, and CHIKV E2 proteins, along with beads coupled to BSA were combined and diluted in PBS-1% BSA-0.02% Tween20 (PBT buffer) at a dilution of 1/200. Fifty μL (containing approximately 1250 beads of each type) of the microsphere suspension were added to each well of black-sided 96-well plates. Serum samples were diluted to 1:400 in PBT buffer. Fifty μL of diluted serum were added to the microspheres in duplicate and incubated for 90 min on a plate shaker set at 700 rpm in a dark biosafety cabinet at room temperature. The plates were washed twice with 200 μL of PBT buffer using a magnetic plate separator (Millipore Corp., Billerica, MA, USA). Fifty μL of red-phycoerythrin (R-PE) conjugated F(ab’)2 fragment rabbit anti-Human IgG (H + L) (Rockland Immunochemicals, Inc., Pottstown, PA, USA) were added at 1 μg/mL to the plates and incubated on a 96-well plate shaker for another 45 min. The plates were washed twice, as above, and microspheres were then resuspended in 120 μL of drive fluid and analyzed on the MAGPIX Instrument (Luminex Corp., Austin, TX, USA). Data acquisition detecting the median fluorescence intensity (MFI) was set to 50 beads per spectral region. Antigen-coupled beads were recognized and quantified based on their spectral signature and signal intensity. The assay background was calculated by averaging the BSA MFI for all samples (US and Liberia separately) and adding three times the standard deviation (shown as grey dotted lines). Unique antigen assay background cutoffs were calculated using known SARS-CoV-2 naive controls collected prior to the COVID-19 pandemic for the Hawaii samples. The average background for each antigen was calculated for all samples, and three standard deviations were added (shown as black lines). Individual antigen backgrounds for Liberian samples were calculated by determining the mean of 20 (~11%) of the serum samples showing the lowest MFI values and adding three standard deviations. Graphical representation of the data was performed using Prism, GraphPad Software v9.5.1 (Boston, MA, USA).

Interpolated IgG concentrations were calculated as previously described [11]. Briefly, SARS-CoV-2 spike reactive IgG was purified by protein A and spike-protein affinity chromatography from pooled sera of humans immunized with SARS-CoV-2 mRNA vaccines. The resulting polyclonal IgG was used to establish standard curves along with the Hawaii and Liberian serum samples suspended in PBT buffer. The standard curves were generated using a two-fold dilution series starting with 5 μg/mL, serially diluted twelve times. Interpolations from MFI to μg/mL were calculated using a sigmoidal dose-response, variable slope model in Prism (GraphPad, Boston, MA, USA). Values below the detection limit were given a value of 0.001 μg/mL. All values determined by interpolation were multiplied by 400 to represent the concentration in the undiluted sample.

### 2.5. Multiplexed Inhibition Tests (MINT)

Microspheres coupled with Wu 1-Trimer, Alpha-S1, and Beta-S1 + S2, were combined and diluted in PBT buffer at a dilution of 1/200. Fifty μL (containing approximately 1250 beads of each type) of the microsphere suspension were added to each well of black-sided 96-well plates. Serum samples were serially diluted in PBT buffer from 1:25 to 1:3200 with doubling dilutions. Fifty μL of diluted serum were added to the microspheres in duplicate and incubated for 90 min on a plate shaker set at 700 rpm in a dark biosafety cabinet at room temperature. The plates were washed twice with 200 μL of PBT buffer using a magnetic plate separator (Millipore Corp., Billerica, MA, USA).

Purified hACE-2 was coupled to R-Phycoerythrin (PE) using the PE/R-Phycoerythrin Conjugation Kit-Lightning-Link^®^ (Abcam ab102918). In total, 100 ng of hACE-2-PE was added to each assay well in a volume of 50 μL and incubated for 2 h on a plate shaker set at 700 rpm in a dark biosafety cabinet at room temperature. The plates were then washed twice with 200 μL of PBT buffer using a magnetic plate separator, and the microspheres were resuspended in 120 μL of drive fluid and analyzed on the MAGPIX Instrument. Data acquisition detecting the median fluorescence intensity (MFI) was set to a minimum of 50 beads per spectral region. Antigen-coupled beads were recognized and quantified based on their spectral signature and signal intensity of hACE-2-PE, respectively.

All samples were run in duplicate and averaged. Averaged MFIs were then normalized to negative control serum MFI to retrieve a percent of inhibition as follows: ((negative control MFI—sample MFI)/negative control MFI) × 100. Percent values were plotted with a dotted line at 50% to indicate MINT_50_ titer cut-offs. Graphical representations plotted with GraphPad Prism.

## 3. Results

### 3.1. MIA Determines SARS-CoV-2 Serological Status

**Figure 1.**
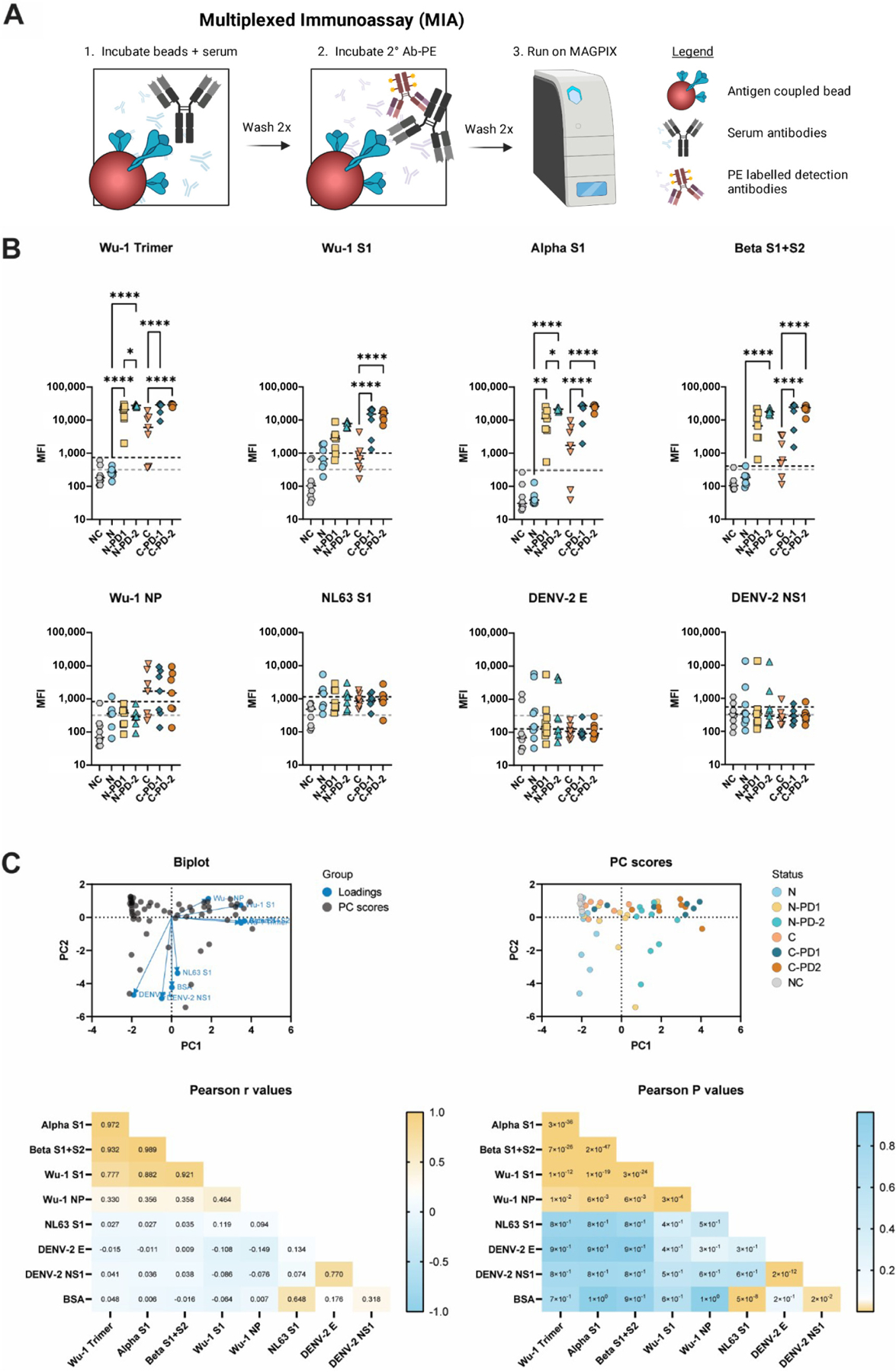
The multiplexed immunoassay (MIA) differentiates SARS-CoV-2 serological status. (**A**) Graphical representation of MIA process created with BioRender.com and Adobe Illustrator. (**B**) MIA of samples collected in Hawaii. One-way ANOVA multiple comparison analysis. Grey lines indicate BSA cut-offs, and black lines indicate antigen cut-offs. (**C**) Principal component analysis of samples collected in Hawaii. Calculations completed in GraphPad Prism, Principal component analysis. * < 0.5, ** < 0.01, **** < 0.0001. 2°Ab-PE = phycoerythrin-coupled secondary antibody, NC = negative controls (n = 10), N = naïve (n = 7), C = convalescent (n = 7), PD1 = post-dose 1, PD2 = post-dose 2. Wu-1 Trimer = Wuhan Hu1 strain spike trimer, Alpha-S1 = SARS-CoV-2 Alpha strain spike domain 1, Beta S1 + S2 = SARS-CoV-2 Beta strain spike domains 1 + 2, Wu-1 NP = Wuhan Hu1 strain nucleocapsid protein, NL63 S1 = hCoV-NL63 spike domain 1, DENV-2E = Dengue virus serotype 2 envelope protein, DENV-2 NS1 = Dengue virus serotype 2 nonstructural protein 1.

To validate the broad MIA panel developed for Liberia, 24 serum samples from a previous longitudinal study investigating antibody responses to COVID-19 vaccines in Hawaii [18] were tested. The samples were stratified into serological groups (naïve or convalescent), as qPCR confirmed in the previous study [18]. The samples were analyzed based on the antigen reactivity of serum IgG using a multiplex immunoassay (MIA) (Figure 1A,B) [8,9,19]. For comparison, the sera collected prior to the SARS-CoV-2 pandemic (n = 10) were analyzed as negative controls (NC). The median fluorescence intensity (MFI) of negative control samples for SARS-CoV-2 antigens were at or below the calculated cut-off values for each antigen (black lines) with some variation using the BSA cut-offs (grey lines) for SARS-CoV-2 Wuhan Hu1 strain nucleocapsid protein (Wu-1 NP), the DENV-2 envelope (DENV-2 E), and non-structural protein 1 (DENV-2 NS1) antigens (Figure 1B). As expected, a stark contrast between the naïve and convalescent samples can be observed for Wuhan-Hu-1 strain spike trimer (Wu-1 Trimer), SARS-CoV-2 Alpha strain spike domain 1 (Alpha S1), Beta strain S1 + S2 (Beta S1 + S2), and Wuhan-Hu-1 NP. Post-dose 1 reactivity trended towards a higher MFI for all SARS-CoV-2 spike variants and became significant after the second dose for all samples. Some of these individuals may have had exposure to the seasonal coronavirus NL63 S1 and DENV-2 antigens based on MFI readings on the respective beads. However, as expected, no changes were observed in reactivities to NL63 nor DENV-2 antigen reactivity through the course of vaccination.

Principal component analysis revealed a clustering of similar antigens into similar quadrants overall and when stratified based on vaccine status (Figure 1C). High Pearson correlation r values were seen between Wu-1 Trimer, Alpha S1, and Beta S1 + S2 antigens, as expected (Figure 1C). Additionally, reactivities to DENV-2 E and DENV-2 NS1 were highly correlated. These correlations were validated with highly significant P-values (Figure 1C). The IgG signatures determined by MIA indicate that this assay can reliably determine SARS-CoV-2 exposure and vaccination status when using the aforementioned assay cut-offs.

### 3.2. Liberian Sera Display Broad Antiviral IgG Reactivity

**Figure 2.**
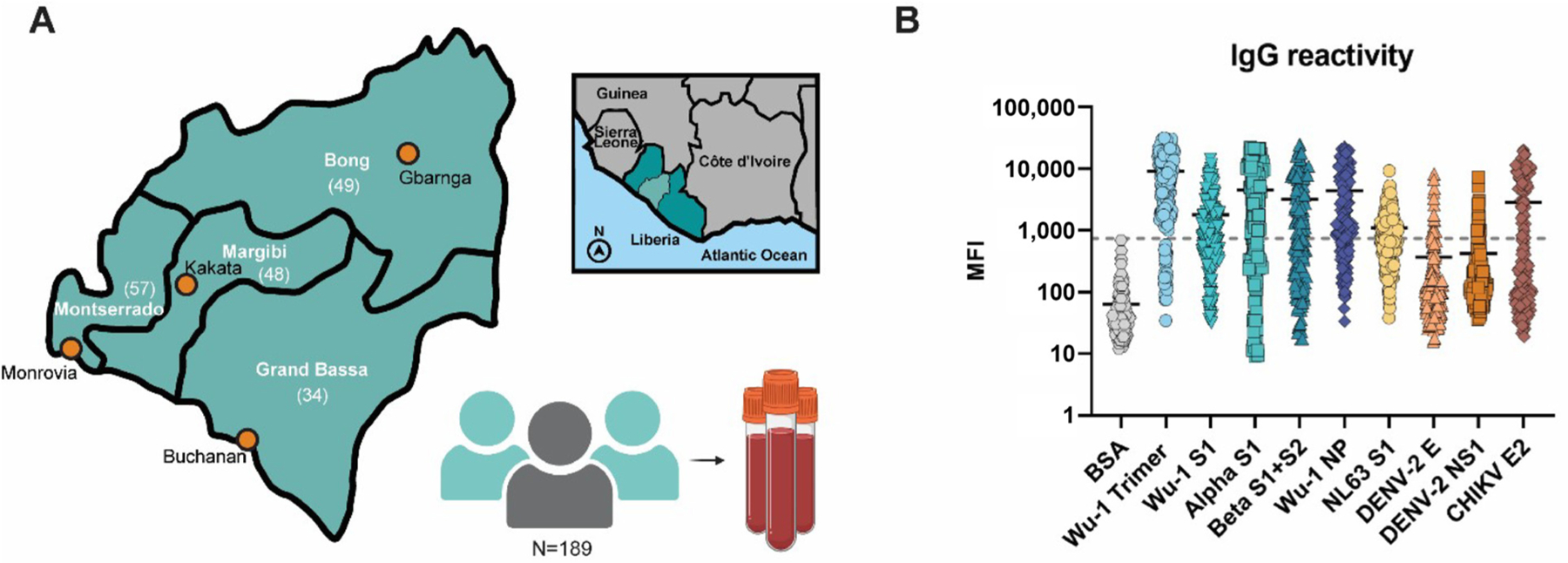
Liberian sera display broad viral antibody reactivity. (**A**) Graphical map indicating locations of the four counties and (n) of the previously collected samples. The four counties are located in central Liberia as indicated by the light green inset. Graphics created with BioRender.com and Adobe Illustrator. (**B**) MIA of overall IgG reactivity. The grey line indicates the cut-off value for the BSA background (mean MFI plus three times the standard deviation).

To investigate the SARS-CoV-2 exposure in Liberia, we sought to determine antigen exposure by using our validated MIA on serum samples that were previously collected in the country (Figure 2). The human sera analyzed originated from Bong (n = 49), Grand Bassa (n = 34), Margibi (n = 48), and Montserrado (n = 57) counties (Figure 2A). The samples display a wide range of IgG reactivity to all antigens in the panel compared to the BSA cut-off (grey line) (Figure 2B). This includes reactivity to antigens of viruses never reported in Liberia, such as hCoV-NL63, DENV-2, and CHIKV.

**Figure 3.**
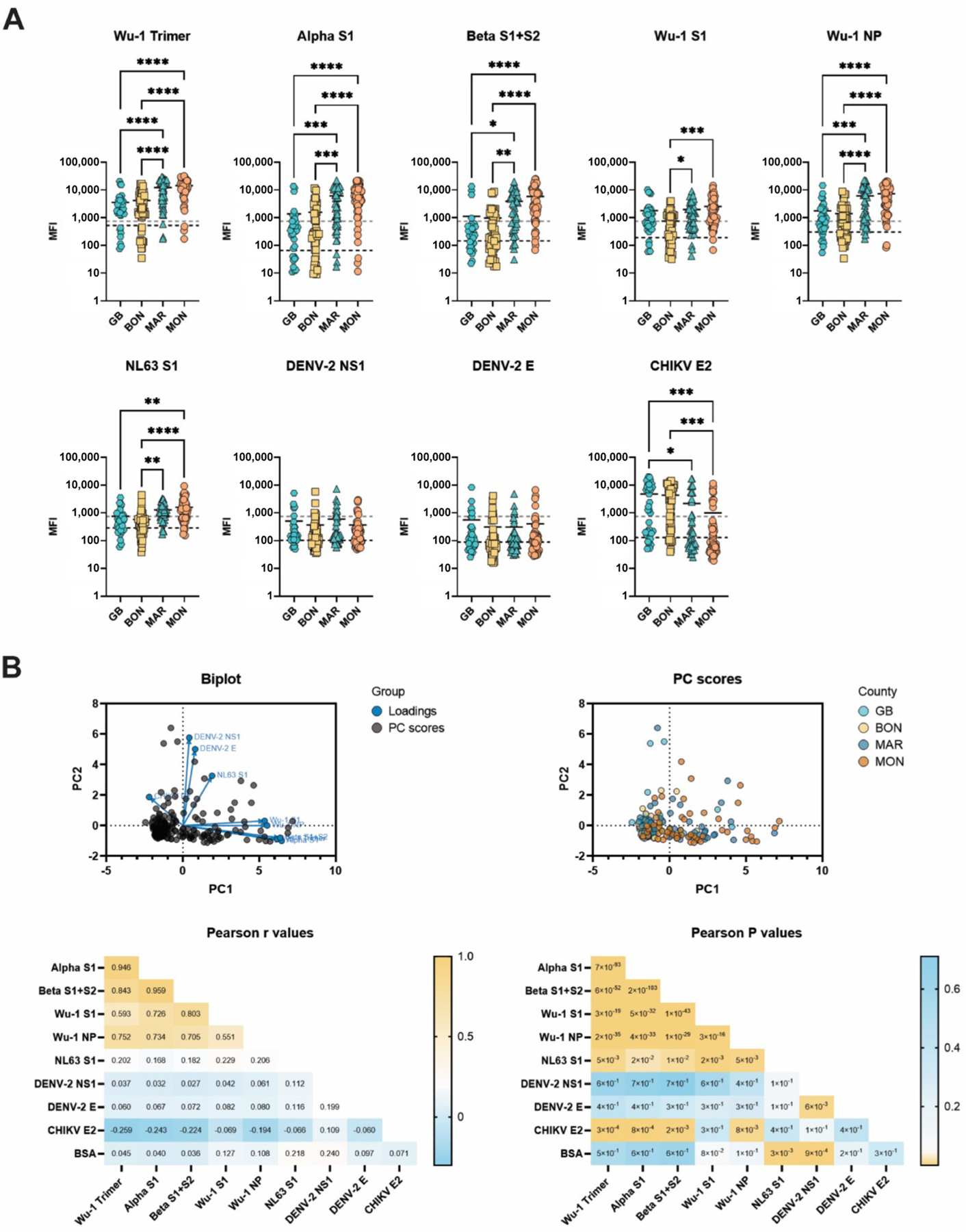
Analysis of serum reactivity in Liberia. (**A**) MIA of samples collected in Liberia. One-way ANOVA multiple comparison analysis. Grey lines indicate BSA cut-offs, and black lines indicate antigen cut-offs. (**B**) Principal component analysis of samples collected in Grand Bassa (GB), Bong (BON), Margibi (MAR), and Montserrado (MON) counties, Liberia (parallel analysis with 1000 simulations). Calculations completed in GraphPad Prism, Principal component analysis. * < 0.5, ** < 0.01, *** < 0.001, **** < 0.0001.

An analysis of IgG reactivity to different antigens revealed that samples from Margibi and Montserrado counties presented the highest reactivities to SARS-CoV-2-related antigens, including NL63 (Figure 3A). Contrarily, a reactivity to CHIKV E2 was higher in the rural Grand Bassa and Bong counties than in the more urban, Montserrado county. Liberian samples display overall higher Wu-1 NP reactivities than the samples collected from naïve and naïve vaccinated individuals in Hawaii. A higher reactivity for NL63 is also noted in Liberia when compared to the Hawaii samples, regardless of SARS-CoV-2 exposure or vaccination status. The reactivities to DENV-2 E, DENV-2 NS1, and CHIKV E2 antigens among Liberian samples are also higher.

A principal component analysis revealed the clustering of reactivity to genetically related antigens, as seen with the Hawaii samples (Figure 3B). Stratification based on county revealed discrete clusters of samples from Margibi and Montserrado counties in the SARS-CoV-2-related antigen sector of the PC score plot. Pearson r values were similar to those calculated with Hawaii samples, revealing high correlations between Wu-1 Trimer, Alpha S1, and Beta S1 + S2 antigens. Wu-1 NP is also highly correlated with other SARS-CoV-2 antigens, a phenomenon generally not seen in the naïve vaccinated samples (Figure 1B), suggesting that a previous SARS-CoV-2 infection can be inferred. Collectively, these results indicate that the MIA assay can also be used to simultaneously determine reactivities to multiple viral antigens when performed in Liberia.

### 3.3. SARS-CoV-2 Spike (Wuhan Hu-1 Strain) Specific IgG Concentrations

**Figure 4.**
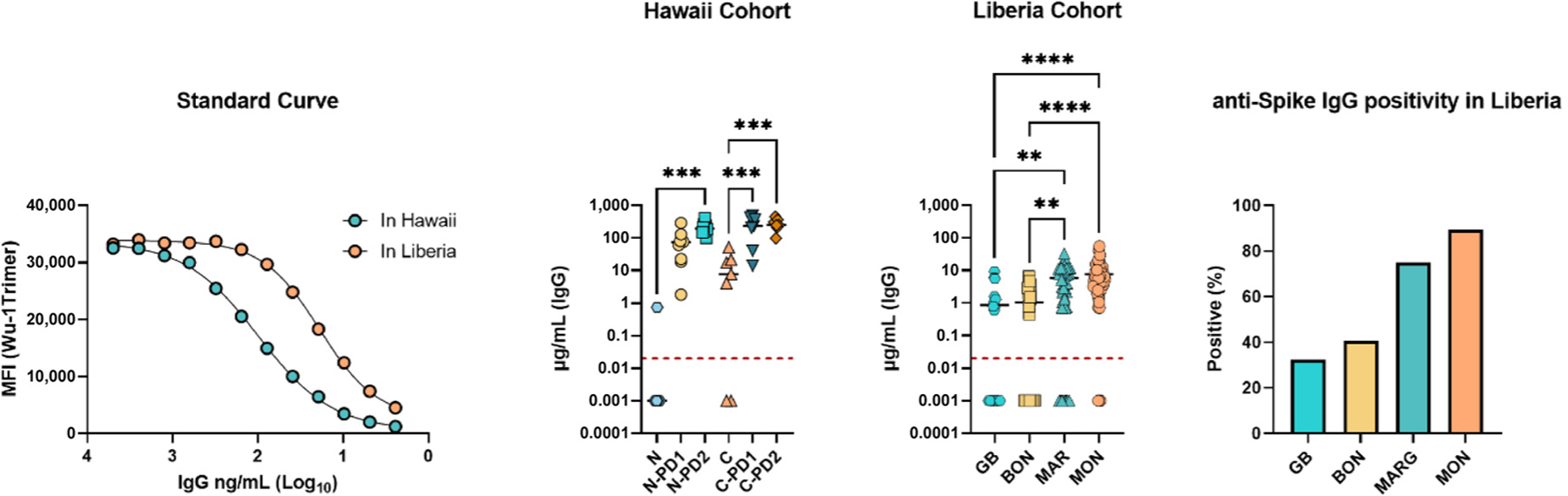
Interpolated anti-Wu-1-Trimer IgG concentrations help to assess serological positivity in Liberia. IgG concentrations were calculated from a standard curve based on polyclonal IgG reactivity to Wu-1 Trimer for samples collected and analyzed in Hawaii or Liberia. The limit of detection is indicated by a red line. Interpolated values that fell below the linear portion of the standard curve were given a value of 0.001 μg/mL for statistical analysis. Sample abbreviations are consistent with Figures 1 and 3. Calculations were performed in GraphPad Prism. ** < 0.01, *** < 0.001, **** < 0.0001.

To determine which samples had a measurable IgG concentration to the Wu-1 Trimer, samples were analyzed simultaneously with a standard curve derived from polyclonal IgG purified from vaccinated individuals [11]. Individual IgG concentrations were calculated by interpolating the raw MFI values of the test samples on a standard curve established using purified polyclonal human anti-Wuhan Hu-1 antibodies. Antigen-specific IgG concentrations rose as expected with an increasing number of vaccine doses in samples collected from either naïve or convalescent persons (Figure 4). Interestingly, one self-reported naïve individual from Hawaii showed a quantifiable concentration of anti-Wu-1 trimer IgG, suggesting a prior infection with SARS-CoV-2 may have gone unnoticed demonstrating the sensitivity of our chosen method. While IgG concentrations were comparable to the convalescent Hawaii samples, unfortunately, no post-vaccination samples were available from Liberia. This analysis also showed a trend of higher seropositivity in urban regions compared to rural ones. This finding was further confirmed when the percentages of samples with detectable IgG concentrations were calculated for each county (% positive), which revealed that samples from the more densely populated Margibi and Montserrado counties showed approximately double the positivity rate measured in samples from Grand Bassa and Bong counties (Figure 4).

### 3.4. Multiplexed Inhibition Test (MINT) Reveals Antibody Functionality

**Figure 5.**
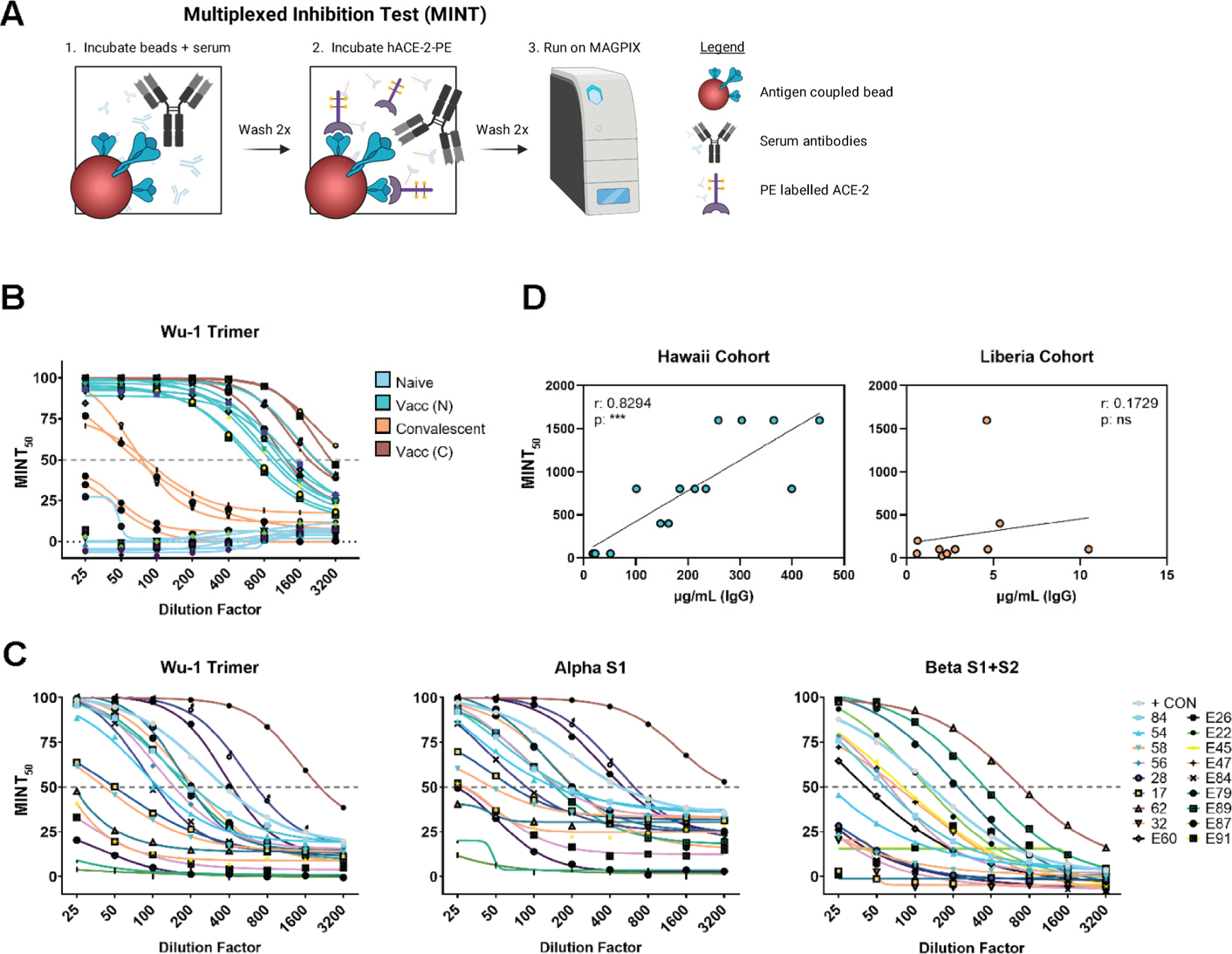
The multiplexed inhibition test (MINT) reveals antibody functionality. (**A**) Graphical representation of the MINT assay created with BioRender.com and Adobe Illustrator. (**B**) MINT curves from Hawaii samples were performed on baseline and post-dose 2 samples for the Wuhan Hu-1 trimer antigen. (**C**) MINT curves for Liberia samples were performed on Wuhan Hu-1 trimer, Alpha-S1, and Beta-S1 + S2 antigens. (**D**) Correlations of MINT_50_ values and interpolated IgG concentrations for samples collected in Hawaii or Liberia. Pearson r and p values were calculated using GraphPad Prism. ***< 0.001.

A multiplexed inhibition test (MINT) was developed to estimate functional immunity in the observed antibody populations without the need for a BSL-3 facility. Functionally, MINT determines the antibody-based inhibition of soluble hACE-2 binding to bead-bound SARS-CoV-2 spike proteins and allows for multiple spike proteins to be tested at once (Figure 5A). The MINT was first tested on the Hawaii samples, separating naïve, convalescent, and post-dose 2 samples into predictable degrees of MINT_50_ (50% inhibition) cut-off scores (Figure 5B). Furthermore, the Pearson r correlation revealed that naïve, convalescent, and post-dose 2 IgG concentrations were highly correlated with MINT_50_ cut-off titers (r = 0.8294, *p* = 0.0001) (Figure 5D). Therefore, Liberian individuals with high, medium, and low reactivities to Wu-1 trimer were selected for MINT. A range of MINT_50_ values was observed against all three variants (Figure 5C). However, when testing a population presenting unknown exposure history or duration from exposure, these values were much less correlative (r = 0.1729, *p* = NS) (Figure 5D) as compared to results from Hawaii samples. Overall, MINT may be useful to determine vaccine status and shed light on antibody functionality in regions where BSL-3 facilities are not available. The assay is adaptable to the MIA platform and enables the detection of antibody-based inhibition of ACE-2 to a SARS-CoV-2 spike protein, in a multiplexed fashion.

## 4. Discussion

The COVID-19 pandemic has prompted global efforts to understand immune responses to SARS-CoV-2 and the rapidly developed and deployed vaccines. While most countries gained rapid access to PCR-based tests and vaccines, Liberia and many parts of Africa remained largely without a supply of these resources in 2021, leaving the reporting on exposure and transmission dynamics limited. Therefore, we developed a portable, multiplexed immunoassay to analyze samples collected from four counties in Liberia. Additionally, we developed a BSL-2 multiplexed inhibition test (MINT) to determine antibody functionality in a region with limited BSL-3 facilities. Our study was conducted at University of Liberia’s Fendall laboratory on samples collected before the major vaccine rollout in Liberia during the summer of 2021. At this time, Liberia and Sierra Leone surged in COVID-19 cases as the Delta variant was detected in the region [20,21]. However, this study did not test for reactivity against Delta variant spike protein as this antigen was unavailable at the time.

The MIA panel could discern between samples collected from naïve, convalescent, and vaccinated individuals in Hawaii. While SARS-CoV-2 spike-specific IgG titers significantly increased after one or two vaccinations, as expected, NP reactivities remained unchanged among naïve and vaccinated naïve study participants. Convalescent individuals, however, did show reactivity to NP, which remained unchanged following vaccination. The group of self-reported naïve individuals contained one person where the MFI and interpolated IgG concentrations may indicate a subclinical infection of SARS-CoV-2 at some point due to reactivities against NP and spike. Additionally, individuals in the Hawaii cohort demonstrating potential exposure to the Dengue virus or a closely related virus, as expected, the reactivity to DENV-2 E and DENV-2 NS1 remained unchanged, including after COVID-19 vaccinations. Furthermore, the variations in BSA and antigen cut-offs highlight the need for conservative and transparent controls when performing multiplexed serological assays. While BSA is commonly used in buffers, it may occasionally serve as an immunogen in individuals that have been exposed to it and mounted a specific response.

We performed a principal component (PC) analysis to validate the reactivity data further. The biplot generated clusters associated with similar antigens, which diverged by quadrants to unrelated antigens. The PC scores indicated predictable clusters of naïve and convalescent samples throughout the vaccination series. As expected by the SARS-CoV-2 reactivity and clustering in the biplot, the spike antigens were highly correlated with significant P-values, while SARS-CoV-2 NP r- values were relatively low but above 0.3 likely due to the convalescent cohort. NL63 S1 reactivity did not correlate with reactivity to SARS-CoV-2 spike antigens, indicating cross-reactivity was an unlikely cause of lower than expected disease burden, despite both viruses using the same cellular receptor for entry. The DENV-2 NS1 and DENV-2 E antigen reactivities were highly correlated and indicated the possibility of natural DENV infection in these particular SARS-CoV-2 naive individuals [22]. The data indicate that our SARS-CoV-2 panel can accurately discern serological status, antigen specificity, and the estimated degree of exposure to SARS-CoV-2 antigens.

The samples from Liberia were collected in June 2021 across Monrovia in Montserrado county, Kakata in Margibi, and the rural areas of Grand Bassa and Bong counties. A wide range of reactivities could be observed for all antigens tested. The samples from Margibi and Montserrado counties showed consistently higher MFI values than those from Grand Bassa and Bong, indicating higher amounts of IgG reactive to the SARS-CoV-2 spike antigens. This regional difference was also seen for NP reactivity, suggesting an increased or more recent viral transmission in the urban regions. The capital city of Monrovia (Montserrado County) and Kakata (Margibi County) are highly populated regions with dense regions of commerce. Urban areas have been reported to be more inundated with SARS-CoV-2 circulation in Liberia and Sierra Leone [20,23]. The results of the MIA assay were able to recapitulate these findings in Liberia. Comparing reactivity to Wu-1 trimer and NP between the Hawaii cohort and Liberian samples revealed that reactivities in Liberia are equivalent to those of convalescent samples collected in Hawaii. Furthermore, we observed a significantly higher correlation between NP and the related SARS-CoV-2 spike antigens in Liberia, which was largely limited to convalescent samples in the Hawaii cohort, as expected. Lastly, the IgG concentrations in a quantifiable range confirmed a Wu-1 Trimer-specific positivity rate of 75% and 89.5% in Margibi and Montserrado counties and 32.4% and 40.8% in Grand Bassa and Bong counties, respectively. Collectively, this likely indicates that a natural infection with SARS-CoV-2 or related viruses, while prevalent in all regions tested, was more common in urban areas.

Interestingly, the urban regions in Liberia also showed significant reactivity to NL63 S1, while no significant titers were observed in any samples collected in Hawaii. Considering reactivities to NL63 S1 remained unchanged during vaccination, NL63 reactivity is likely not caused by cross-reactivity. Although not previously reported in Liberia, seasonal coronaviruses (sCoVs) likely circulate in the region [24,25]. The sCoVs present in the region and to what degree their presence may impact the seroreactivity to SARS-CoV-2 in Liberia is not known. Although infection with sCoVs provides only short-term protection from reinfection (~12 months) [26], it is still unclear to what degree this may affect immunity to SARS-CoV-2. It has been suggested that cross-protection can occur but cannot explain the patterns of SARS-CoV-2 age-dependent susceptibility [27]. Other studies indicate cross-protection may depend on IgG levels against sCoVs at the time of SARS-CoV-2 infection [28,29]. Exposure to sCoVs NL63, OC43, and 229e has been reported in neighboring Sierra Leone with positive correlations to SARS-CoV-2 N protein reactivities [30]. However, the extent of infection or reinfection of sCoVs in Liberia is unknown. Therefore, Liberia may be a new frontier to further investigate the immunological implications of sCoV and SARS-CoV-2 infections.

While Dengue viruses have been reported in neighboring Sierra Leone and Cote d’Ivoire [31–34], evidence of Dengue viruses in Liberia remains poorly reported. However, the primary mosquito vector for Dengue viruses, *Aedes aegypti*, is known to be present in Liberia, and Dengue viruses are likely to be circulating within the country [34,35]. While we did find evidence of reactivity to DENV-2 envelope and DENV-2 NS1 in Liberia, with this assay, we could not determine which serotypes may be present or how prevalent these viruses may be. No significance was reached between counties indicating that DENV or a closely related flavivirus may be widespread within Liberia. While our findings support the probability of Dengue viruses being present in the region, further work is needed to detail the prevalence and geographic distribution of flaviviruses in the country.

Another virus spread by *Aedes aegypti*, CHIKV, has also been reported in neighboring Sierra Leone and Guinea [32,36], but so far, reporting in Liberia remains limited. While reports for arboviruses remain overall limited throughout the region, we did see evidence of CHIKV within the country, based on the observed CHIKV E2 reactivities. In opposition to SARS-CoV-2 reactivities, the more rural regions of the Grand Bassa and Bong counties showed significantly higher reactivities to CHIKV E2 than the urban regions. This finding could be due to the greater proximity of other primates to humans in rural regions within Liberia. The overall detection of arboviruses in Liberia highlights the need to study the region further to better understand its viral landscape.

Several ELISA-based SARS-CoV-2 inhibition assays have been developed to determine surrogate neutralization without the need for BSL-3 laboratories [15,37–39]. We developed an inhibition assay to complement our MIA panel and capitalize on the multiplexed functionality of bead-based systems. We first tested the MINT with the Hawaii cohort samples. The test was able to predictively segregate naïve and convalescent samples from post-dose two immunized samples with spike-trimer coated beads. Furthermore, we saw a high correlation between IgG concentrations and MINT_50_ titers among the Hawaii cohort samples, which has been previously demonstrated to correlate with neutralization titers [40–43]. We next employed the system for low, medium, and high-titer individuals in Liberia. We saw varying degrees of inhibition among the samples, which did not reach any significant correlations to total IgG reactivity. This may be due to the variability in natural infection, the duration since infection, and functional antibodies of isotypes other than IgG. Additionally, individuals that show high IgG titers in response to a natural infection with SARS-CoV-2 or a related virus may not have developed neutralizing antibodies, and conversely, individuals with low IgG titers may have produced potent neutralizing antibodies that are not abundant. We showed with our experiments that this multiplexed test could predictably determine antibody functionality (inhibition of cell-receptor binding) rapidly and for multiple variants in a single well. This test would be ideal for field studies in which selecting samples of interest will help accelerate further study.

Collectively, bead-based serological assay systems enable the multiplex detection of exposure to numerous relevant pathogens. Although these adaptable assays cannot determine active infections, they hold potential as valuable tools for LMICs, especially in areas with limited cold-chain storage capabilities. In this study, the Luminex Magpix system was chosen for its affordability and portability. Moreover, the recombinant antigen coupled MagPlex^®^ microspheres demonstrate resilience to degradation in unstable temperature conditions [44]. It has been observed that Luminex may be transitioning away from these units, possibly favoring more expensive and less portable configurations. Furthermore, previously available laptops have been replaced with desktop units. Such changes could pose challenges for assay support in LMICs if reliant on Luminex technologies. The development of advanced, cost-effective, and portable multiplexed assay technologies might be essential for future pandemic preparedness in LMICs.

This study aimed to implement a multiplexed platform to determine SARS-CoV-2 exposure in Liberia. We observed indications of widespread natural infection in the region with surprisingly high seropositivity percentages. Concurrently, we could identify largely unreported exposure to hCoV-NL63, as well as reactivity to DENV and CHIKV, potentially endemic arboviruses for which this region lacks prevalence data. We further implemented a multiplexed inhibition test (MINT) to determine the serological inhibition of hACE-2 binding to several SARS-CoV-2 variant spike proteins as an indicator of functional antibody responses in these sera. These two multiplexed assays are complementary, relatively easy to transport and, therefore, maximize data output for field research.

## Supplementary Material

Supplementary Figure S1

## Data Availability

Data will be provided upon reasonable request by the corresponding author, Axel T. Lehrer (lehrer@hawaii.edu).
