# Supplementary material for: A Serological Multiplexed Immunoassay (MIA) Detects Antibody Reactivity to SARS-CoV-2 and Other Viral Pathogens in Liberia and Is Configurable as a Multiplexed Inhibition Test (MINT)
Source: Immuno. Author manuscript; Available in PMC 2024 Oct 10. (PMC11465787; doi:10.3390/immuno4010007)
Supplement: Supplementary Figure S1 [file NIHMS2026013-supplement-Supplementary_Figure_S1.pdf]

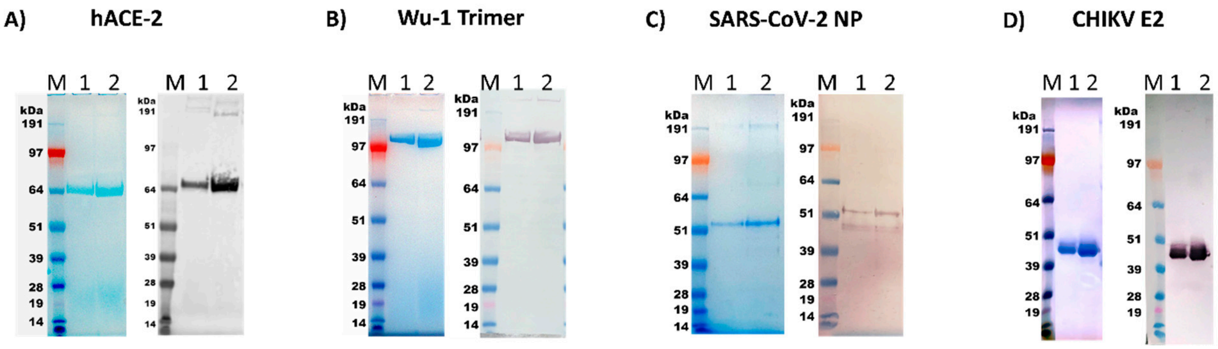

Figure S1. SDS-PAGE Coomassie Stains and Western blots of Purified, Drosophila S2 cell-derived, recombinant proteins of A) hACE-2, B) Wu-1 Trimer, C) SARS-CoV-2 NP and D) CHIKV E2. Western blots were detected with 6x-his tag monoclonal antibody (1:10,000 dilution), mAb CR3022 (1:1000 dilution), human SARS-CoV-2 convalescent sera (1:500 dilution) and human CHIKV convalescent sera (1:500 dilution), respectively. Purity of each protein based on the SDS-PAGE is estimated to be about >80%, >90%, ~60%, and >90%, respectively. M: Marker, 1: 1 μg, and 2: 2 μg of protein.
